# Robotic approach together with an enhanced recovery programme improve the perioperative outcomes for complex hepatectomy

**DOI:** 10.3389/fsurg.2023.1135505

**Published:** 2023-06-02

**Authors:** Fei Xie, Dongdong Wang, Jin Ge, Wenjun Liao, Enliang Li, Linquan Wu, Jun Lei

**Affiliations:** ^1^ Department of General Surgery, Second Affiliated Hospital of Nanchang University, Nanchang, China; ^2^ Jiangxi Province Engineering Research Center of Hepatobiliary Disease, Second Affiliated Hospital of Nanchang University, Nanchang, China

**Keywords:** enhanced recovery after surgery, outcomes, complex liver resection, robotic surgery, eras

## Abstract

**Objective:**

Robotic surgery has more advantages than traditional surgical approaches to complex liver resection; however, the robotic approach is invariably associated with increased cost. Enhanced recovery after surgery (ERAS) protocols are beneficial in conventional surgeries.

**Methods:**

The present study investigated the effects of robotic surgery combined with an ERAS protocol on perioperative outcomes and hospitalization costs of patients undergoing complex hepatectomy. Clinical data from consecutive robotic and open liver resections (RLR and OLR, respectively) performed in our unit in the pre-ERAS (January 2019–June 2020) and ERAS (July 2020–December 2021) periods were collected. Multivariate logistic regression analysis was performed to determine the impact of ERAS and surgical approaches—alone or in combination—on LOS and costs.

**Results:**

A total of 171 consecutive complex liver resections were analyzed. ERAS patients had a shorter median LOS and decreased total hospitalization cost, without a significant difference in the complication rate compared with the pre-ERAS cohort. RLR patients had a shorter median LOS and decreased major complications, but with increased total hospitalization cost, compared with OLR patients. Comparing the four combinations of perioperative management and surgical approaches, ERAS + RLR had the shortest LOS and the fewest major complications, whereas pre-ERAS + RLR had the highest hospitalization costs. Multivariate analysis found that the robotic approach was protective against prolonged LOS, whereas the ERAS pathway was protective against high costs.

**Conclusions:**

The ERAS + RLR approach optimized postoperative complex liver resection outcomes and hospitalization costs compared with other combinations. The robotic approach combined with ERAS synergistically optimized outcome and overall cost compared with other strategies, and may be the best combination for optimizing perioperative outcomes for complex RLR.

## Introduction

Hepatectomy is an important treatment option for benign and malignant liver tumors. The difficulty of this surgery varies according to the location, size, relationship between adjacent blood vessels and bile ducts, and congenital anatomical variation of the tumor(s). Several studies have shown that the difficulty of liver resection directly correlates with the incidence of postoperative complications (1, 2).

Minimally invasive surgery has emerged in recent decades; its advantages over open surgery include smaller incisions, less postoperative pain, a shorter LOS, and less bleeding. Robotic surgery is the most recently developed element of minimally invasive surgery. While retaining most of the advantages of laparoscopic surgery, it also offers magnified three-dimensional high-resolution views, flexible wrist instruments, and motion and tremor filtering (3). Given its improved ergonomics, robotic surgical systems may be more suitable for controlling intraoperative bleeding, performing miniscule suture and vascular separation procedures, ensuring adequate surgical margins, and performing complex liver resections with a high difficulty level (4, 5). Some studies have reported that robotic surgery can reduce postoperative complications associated with complex surgeries and intensive care unit admission rates (6–8). Our previous studies have found that, for complex liver resection, robotic surgery is superior to laparoscopic and open surgery in terms of conversion rates and incidence of serious complications. However, robotic liver resection (RLR) is often more expensive than traditional/conventional [i.e., open liver resection (OLR)] surgery.

Enhanced recovery after surgery (ERAS) protocols are care programs designed to minimize postoperative stress and accelerate postoperative recovery through standardized multimodal perioperative management. Their core elements include fluid management, pain control, early oral intake, and the promotion of early mobilization and recovery after surgery. Over the past several decades, the implementation of ERAS protocols has improved outcomes in various surgical specialties, including colorectal (9), urological (10, 11), and bariatric surgeries (12), while reducing complication rates, the length of hospital stay (LOS), and costs.

Based on successful perioperative experience with ERAS protocols and the characteristics of robotic minimally invasive surgery, RLR + ERAS appears to be a feasible solution to optimize postoperative outcomes through enhanced perioperative management for complex liver surgery. Several retrospective reviews have shown that ERAS protocols, in combination with robotic surgery, can significantly reduce LOS and patient costs (8, 13–16). In this study, we combined a robotic surgical system with ERAS to investigate the impact on perioperative outcomes and hospital costs among patients undergoing complex hepatectomy.

## Methods

### Study design

The complexity of hepatectomy was scored based on the IWATE criteria proposed at the Second International Consensus Conference on Laparoscopic Liver Resections held in Morioka, Japan, in 2014. The total IWATE score was calculated as the sum of the following six difficulty measures: tumor location (score, 1–5), extent of hepatic resection (score, 0–4), tumor size (score 0 or 1), proximity to a major vessel (score 0 or 1), liver function (score 0 or 1), and HALS/hybrid (score 0 or −1). The 12 difficulty levels were divided into four types: low (0–3), intermediate (4–6), advanced (7–9) and expert (10–12) (17, 18).

According to the IWATE scoring criteria, advanced and expert grades were defined as complex hepatectomy. A retrospective study was performed to collect clinicopathological data from 171 patients at our center before (January 2019–June 2020) and after the implementation of the complex liver resection-specific ERAS protocol (July 2020–December 2021). The ERAS protocol was implemented in July 2020 and uses current evidence-based guidelines for the perioperative management of liver resection (Supplementary Table S1). Complex hepatectomy was performed by four surgeons, two of whom performed rigorous open hepatectomy and two performed both open and robotic hepatectomy. According to ERAS implementation, patients were divided into pre-ERAS and ERAS cohorts as well as open and robotic surgical cohorts based on surgical methods.

**Figure 1:**
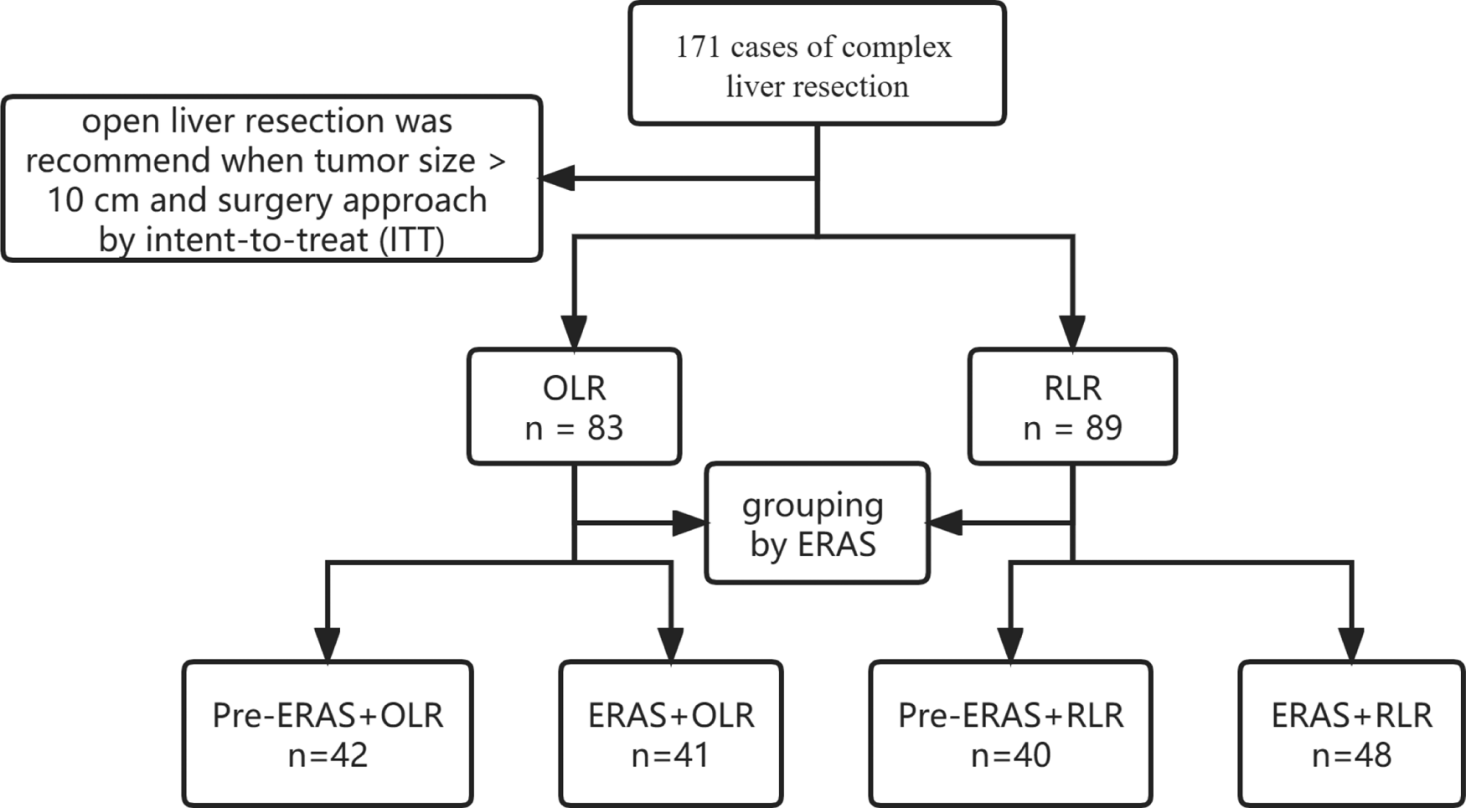
Flow diagram of the study.

The surgical methods for all hepatectomy cases in this study were selected on an intent-to-treat basis. On the one hand, surgeons decide on surgical procedures based on the characteristics of the tumor and the patient's fitness. In general, robotic surgery is considered for patients with a tumor size <10 cm who do not need additional vascular or bile duct resection and reconstruction. On the other hand, all the patients were preoperatively informed of their surgeon's experiences in open and robotic liver resection, the present situation and expected surgical results of each operative approach, the potential advantages and disadvantages of robotic liver resection, and the possibility of a conversion of laparotomy. The patient selected from the two surgical methods after a thorough discussion of the advantages and limitations (Figure 1).

All patients were generally in good condition, with preoperative Child‒Pugh A or B class liver function or an indocyanine green clearance (15 min) rate <15%. Furthermore, the patients were free of distant lymph node metastasis, adjacent organ invasion, or distant organ metastasis. All patients were examined through multidisciplinary consultation before surgery, including surgery, medical oncology, hepatology, and imaging experts. The clinicopathological data were complete. Patients were informed about the operative details of the treatment, including surgical procedures, risks, and complications. They provided written informed consent to undergo treatment. The ethics committee of our unit approved the research program and supervised the research process.

### Primary outcomes and definitions

General and perioperative data were collected from all patients. The general information and clinical characteristics of the patients included age, sex, body mass index, American Society of Anesthesiologists (ASA) grade, preoperative liver function score, degree of cirrhosis, hypertension and diabetes. Perioperative data included operative duration, intraoperative blood loss, postoperative complications, LOS, and hospitalization cost. The primary outcome was length of stay (LOS), and the secondary outcomes included postoperative complication rate and total hospital cost.

Surgical duration was defined as the interval from the beginning of skin incision to the end of hepatectomy, abdominal cavity closure, and skin suturing; the surgical duration of the robotic approach included the time for dock and undocking of the robotic arm.

A visual analog scale (VAS) was used to evaluate patient pain levels, with a score ≥ 4 defined as pain requiring analgesic treatment. Multimodal analgesia: 40 mg ParecoxibNa/Flurbiprofen i.v. per 12 h and 400 mg ibuprofen/diclofenac sodium (50–100 mg) twice a day orally. PCA was used if necessary (flurbiprofen/bupivacaine/ropivacaine plus low-dose dezocine). Where possible, other effective analgesics should be used to replace opioids for analgesia. Intraoperative anesthesia induction included the opioid sufentanil (0.5 µg/kg). Intraoperative analgesia maintenance: Analgesia was adjusted according to the analgesia nociception index (ANI). With ANI  >  60, sufentanil was injected intravenously at 0.1 µg/kg. Postoperative PCA contained 15 mg dezocine. All postoperative complications were assessed within 30 days of surgery and were recorded and classified according to the Clavien–Dindo system (19). Major complications were defined as any complication requiring an invasive procedure, surgery, or admission to the intensive care unit, and those resulting in death (Clavien–Dindo grade III–V).

### Statistical analysis

Normally distributed continuous variables are expressed as the mean ± standard deviation (SD); nonnormally distributed data are expressed as the median and interquartile range (IQR), and categorical data are expressed as frequencies and percentages. The chi-squared test or Fisher's exact test was used to compare categorical variables, Student's *t* test was used to compare normally distributed continuous variables, and nonparametric Kruskal–Wallis and Wilcoxon rank-sum tests were used for nonnormally distributed data. Based on clinical importance, scientific knowledge, and previously published articles, we collected clinical variables that might be related to postoperative LOS and cost, including ERAS protocol, surgical approach, age, ASA classification, cirrhotic liver and Child‒Pugh class. Univariate and multivariate analyses were then performed to identify risk factors associated with increased postoperative LOS and cost, whereas multivariate logistic regression analysis was performed to determine the effects of ERAS, surgical approach, and combinations of ERAS surgical approaches on LOS and cost. A forest diagram drawn by R version 4.0.3 was used to demonstrate the results of univariate and multivariate regression analyses. Statistical analysis was performed using Statistical Package for the Social Sciences version 20.0 (IBM Corporation, Armonk, NY, USA). Differences with *P *< 0.05 were considered to be statistically significant.

## Results

### Clinical characteristics of the liver resection patients

Clinicopathological data from 171 consecutive patients who underwent complex liver resection were included in this study (Table 1). The median patient age was 62 years, and 66% were male. Fifty-one percent of patients underwent liver resection using the robotic approach, and 86% had Child‒Pugh class A liver function. The primary comorbidities included cirrhosis (45%), diabetes (11%), and hypertension (14%). The overall postoperative complication rate was 35%, and the incidence of serious complications (Clavein-Dindo grade > 2) was 11%. The median LOS was 8 days, and the average hospitalization cost was 59,609 CNY. Based on the ERAS protocol and surgical approach, all patients were divided into four groups as follows: pre-ERAS + OLR (*n* = 42); pre-ERAS + RLR (*n* = 40); ERAS + OLR (*n* = 41); and ERAS + RLR (*n* = 48) (Table 1). In the ERAS + OLR group, compliance with the ERAS protocol was 80%, while in the ERAS + RLR group, compliance was 85%. Our results showed that patients in each group were evenly matched in preoperative variables (Table 1).

**Table 1:** Preoperative characteristics of liver resection patients.

	Total (*n* = 171)	Pre-ERAS + OLR (*n* = 42)	Pre-ERAS + RLR (*n* = 40)	ERAS + OLR (*n* = 41)	ERAS + RLR (*n* = 48)	*P*
Age (years)	61.8 ± 12.3	63.5 ± 13.8	60.8 ± 11.5	60.3 ± 11.4	62.3 ± 13.3	0.6435
Sex, Male/Female	113/58	27/15	23/17	31/10	32/16	0.3851
BMI (kg/m^2^)	25.2 ± 6	26.1 ± 5.8	24.1 ± 6.3	24.2 ± 6.2	26.3 ± 5.4	0.1628
ASA classification						0.5434
≤ 2	152 (88%)	36 (85%)	36 (90%)	35 (85%)	45 (94%)	
> 2	19 (12%)	6 (15%)	4 (10%)	6 (15%)	3 (6%)	
Albumin (g/L)	38.4 ± 5.8	38.9 ± 5.4	38.1 ± 6	37.1 ± 6.2	39.1 ± 5.4	0.3618
TBIL (μmol/L)	19.1 ± 11.7	20.1 ± 10.8	18.1 ± 12.3	18.4 ± 12.4	19.6 ± 11.3	0.8412
ICG-R15min (%)	10.2 ± 6.5	10.4 ± 6.1	10.1 ± 6.6	9.7 ± 6.8	10.4 ± 6.3	0.9528
Cirrhotic liver, *n* (%)	77 (45%)	17 (40%)	19 (47%)	23 (56%)	18 (37%)	0.3107
Hypertension	24 (14%)	8 (19%)	4 (10%)	5 (12%)	7 (14%)	0.6727
Diabetes	19 (11%)	5 (12%)	6 (15%)	5 (12%)	3 (6%)	0.6070
Child-Pugh class, *n* (%)						0.6416
A	148 (86%)	34 (80%)	35 (87%)	37 (90%)	42 (87%)	
B	23 (14%)	8 (20%)	5 (13%)	4 (10%)	6 (13%)	
Hepatic segments involved by resected tumor (*n*)						1
1	13	2	3	4	4	
2	37	8	9	11	9	
3	38	8	9	11	10	
4A	62	16	15	13	18	
4B	64	18	17	14	15	
5	46	9	8	13	16	
6	51	10	16	14	11	
7	42	12	9	7	14	
8	50	9	11	13	17	
ERAS compliance	-	-	-	33(80%)	41(85%)	0.5358

*Bold values indicate statistically significant *p*-value (*p* < 0.05).

ASA, American Society of Anesthesiologists; BMI, body mass index; ICG, indocyanine green; TBIL, total bilirubin.

### Perioperative outcomes of the liver resection patients

Among perioperative outcomes, compared with the pre-ERAS cohort, liver resection in the ERAS era was associated with earlier postoperative first ambulation (Pre-ERAS vs. ERAS, 2.2 ± 1.2 days vs. 1.3 ± 0.8 days; *P *< 0.0001), earlier mean postoperative return of bowel function (Pre-ERAS vs. ERAS, 2.7 ± 0.9 days vs. 1.8 ± 1.0 days; *P *< 0.0001), and less postoperative pain (Pre-ERAS vs. ERAS, 4.6 ± 1.5 vs. 2.5 ± 1.2; *P *< 0.0001) (Table 2). The incidences of total postoperative or major complications did not differ between the two groups. The median LOS (pre-ERAS vs. ERAS, 8 vs. 7 days; *P *= 0.001) and median total hospitalization costs (pre-ERAS vs. ERAS, 61,105 CNY vs. 57,886 CNY, *P *= 0.0366) were significantly lower in the ERAS cohort than in the pre-ERAS cohort.

**Table 2:** Operative characteristics and postoperative outcomes of liver resection patients.

	Total (*n* = 171)	Pre-ERAS (*n* = 82)	ERAS (*n* = 89)	*P*	OLR (*n* = 83)	RLR (*n* = 88)	*P*
Operative time, min	229.7 ± 112.5	223.8 ± 116.3	236.9 ± 108.9	0.4479	195.3 ± 97.5	260.7 ± 116.3	**0** **.** **0001** *
Intraoperative blood loss, mL	249.8 ± 152.1	266.3 ± 148.6	236.7 ± 167.8	0.2253	362.5 ± 176.3	216.6 ± 138.7	**0** **.** **0001** *
POD first ambulation, day	1.8 ± 1	2.2 ± 1.2	1.3 ± 0.8	**0** **.** **0001** *	2.3 ± 1.3	1.4 ± 0.8	**0** **.** **0001** *
Pain score	3.5 ± 1.3	4.6 ± 1.5	2.5 ± 1.2	**0** **.** **0001** *	5 ± 1.5	2 ± 1.2	**0** **.** **0001** *
Return of bowel function, day	2.3 ± 1	2.7 ± 0.9	1.8 ± 1.0	**0** **.** **0001** *	2.6 ± 1.2	1.9 ± 0.8	**0** **.** **0001** *
LOS, day	8 (6–9)	8 (7–10)	7 (6–9)	**0** **.** **001** *	9 (7–10)	7 (6–9)	**0** **.** **0001** *
Total postoperative Complication (%)	61 (35%)	34 (41%)	27 (30%)	0.1292	35 (42%)	26 (29%)	0.085
Clavien I–II (%)	42 (24%)	24 (29%)	18 (20%)	0.1699	20 (24%)	22 (25%)	0.8909
Clavien III–IV (%)	19 (11%)	10 (12%)	9 (10%)	0.665	15 (18%)	4 (4%)	**0** **.** **0065** *
Hospital cost (CNY)	59,609 (52,330–65,530)	61,105 (55,305–68,230)	57,886 (51,623–63,914)	**0** **.** **0366** *	57,878 (48,550–64,325)	60,678 (53,350–68,235)	**0** **.** **011** *

LOS, length of hospital stay; POD, postoperative day.

* Bold values indicate statistically significant *p*-value (*P* < 0.05).

Compared with the open approach, RLR had a longer operative duration (OLR vs. RLR, 195.3 ± 97.5 min vs. 260.7 ± 116.3 min; *P *< 0.0001) but had decreased intraoperative blood loss (OLR vs. RLR, 362.5 ± 176.3 ml vs. 216.6 ± 138.7 ml; *P *< 0.0001), a lower pain score (OLR vs. RLR, 5 ± 1.5 vs. 2 ± 1.2; *P *< 0.0001), earlier postoperative first ambulation (OLR vs. RLR, 2.3 ± 1.3 days vs. 1.4 ± 0.8 days; *P *< 0.0001) and decreased median LOS (OLR vs. RLR, 9 vs. 7 days; *P *< 0.0001). The incidence of postoperative complications did not differ among the four groups; however, the incidence of postoperative Clavien III–IV complications was significantly lower in the RLR cohort than in the OLR cohort (OLR vs. RLR, 8.7% vs. 2.3%; *P *= 0.0065). The overall incidence of postoperative complications did not significantly differ between OLR + ERAS and RLR + ERAS (OLR + ERAS vs. RLR + ERAS, 34% vs. 27%; *P* = 0.47), and the incidence of postoperative Clavien III–IV complications did not significantly differ between the two groups (OLR + ERAS vs. RLR + ERAS, 17% vs. 4%; *P* = 0.0746). These results suggest that robotic complex hepatectomy combined with ERAS can achieve the same results as open complex hepatectomy and is even better than open hepatectomy in terms of the incidence of serious complications. Compared with OLR, the median hospitalization cost (OLR vs. RLR, 57,878 CNY vs. 60,678 CNY; *P *= 0.011) was higher in patients who underwent RLR (Table 2).

In addition, further analysis was performed and revealed that patients in the ERAS + RLR group recovered more quickly than those in the other groups, manifested as earlier postoperative first ambulation, less postoperative pain and earlier postoperative return of bowel function (Table 3). Compared to the other groups, the ERAS + RLR group had the shortest postoperative median LOS (6 days, *P* = 0.0001), and it had the lowest incidence of postoperative major complications compared to open surgery (*P* = 0.0458). Among the 4 groups, the total hospitalization cost of patients in the pre-ERAS + RLR group was the highest, and the total hospitalization cost did not significantly differ between the ERAS + RLR group and the open surgery group. Detailed costs are presented in Supplementary Table S2.

**Table 3:** Outcomes by ERAS and operative approach.

	Total (*n* = 171)	Pre-ERAS + OLR (*n* = 42)	Pre-ERAS + RLR (*n* = 40)	ERAS + OLR (*n* = 41)	ERAS + RLR (*n* = 48)	*P*
Operative time, min	229.7 ± 112.5	193.7 ± 120.8	252.7 ± 110.6	232.6 ± 93.7	273.2 ± 120.8	**0.0066** *
Intraoperative blood loss, ml	249.8 ± 152.1	386.2 ± 150.7	206.1 ± 140.1	359.6 ± 187.5	229.5 ± 110.7	**0.0001** *
POD first ambulation, day	1.8 ± 1	2.5 ± 1.4	1.5 ± 0.8	2 ± 1.1	1.2 ± 0.7	**0.0001** *
Pain score	3.5 ± 1.3	5.9 ± 1.8	2.2 ± 1.3	3.6 ± 1.4	1.7 ± 0.8	**0.0001** *
Return of bowel function, day	2.3 ± 1	2.9 ± 1.3	2.2 ± 0.8	2.5 ± 1.1	1.6 ± 0.7	**0.0001** *
Hospital LOS, day	8 (6–9)	9 (7–11)	8 (6–9)	8 (7–9)	6 (5–8)	**0.0001** *
Total postoperative Complication(%)	61 (35%)	21 (50%)	13 (32%)	14 (34%)	13 (27%)	0.1376
Clavien I–II(%)	42 (24%)	13 (30%)	11 (27%)	7 (17%)	11 (27%)	0.4893
Clavien Ⅲ–Ⅳ (%)	19 (11%)	8 (19%)	2 (5%)	7 (17%)	2 (4%)	**0.0458** *
Hospital Cost(CNY)	59,609 (52,330–65,530)	58,787 (49,856–63,235)	64,334 (57,968–72,458)	54,892 (45,510–63,914)	58,772 (52,516–63,241)	**0.0064** *

LOS, length of hospital stay; POD, postoperative day.

* Bold values indicate statistically significant *p*-value (*p *< 0.05).

### Analysis of the cause of prolonged LOS after LR

**Figure 2:**
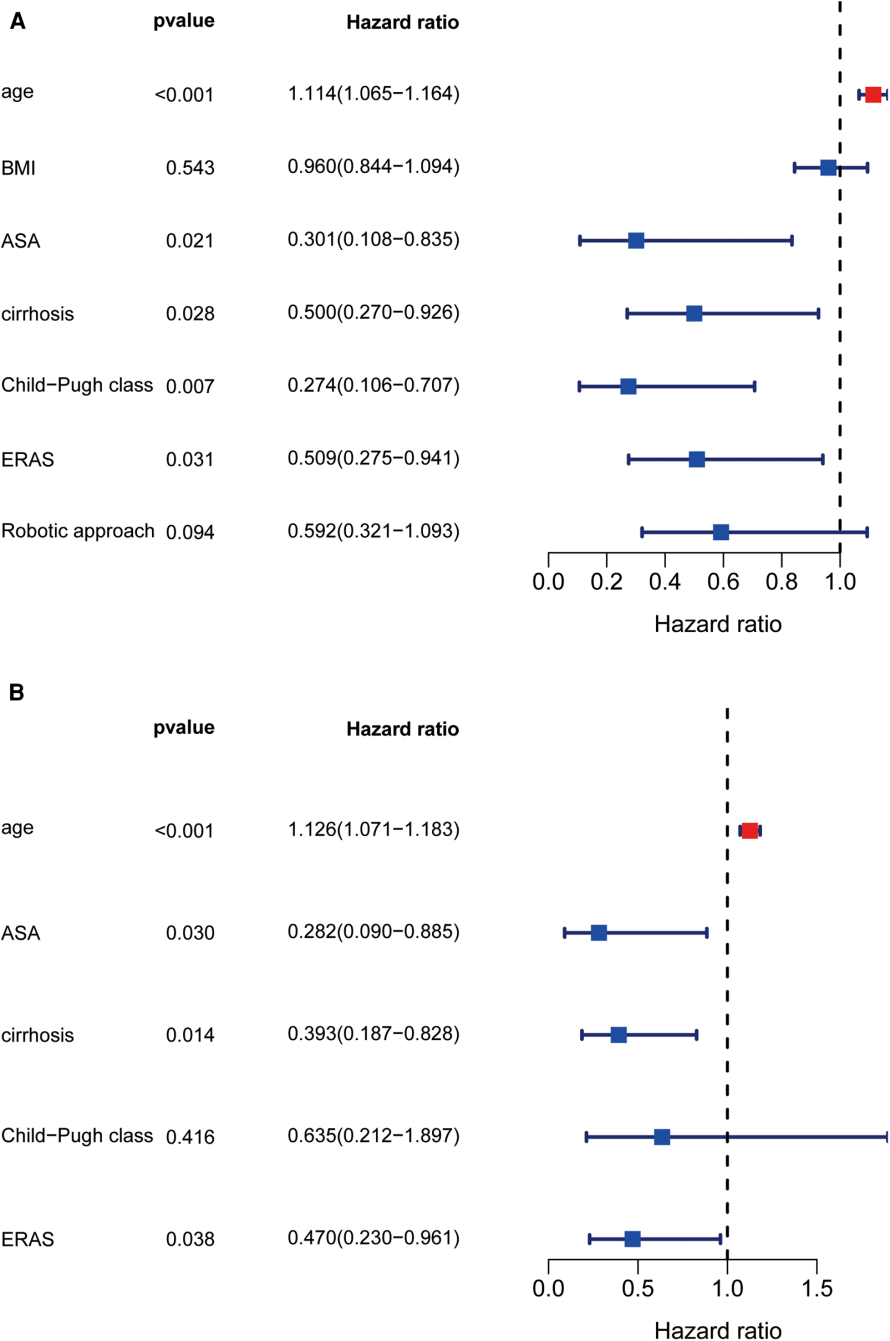
Univariate and multivariate regression analysis about prolong LOS. Forest diagram showed the results of Univariate and multivariate regression analysis (**A,B**).

On univariate analysis (Figure 2A), factors associated with prolonged LOS included increased age, ASA classification, cirrhosis, preoperative Child‒Pugh class B liver function, non-ERAS protocol, and OLR approach. Multivariate analysis revealed that age (odds ratio [OR]: 1.112 [95% confidence interval (CI): 1.061–1.167]; *P *< 0.0001) and cirrhosis [OR: 0.368 (95% CI: 0.17–0.798); *P *= 0.011] remained independently associated with prolonged LOS, while the robotic approach and ERAS pathway [OR: 0.345 (95% CI: 0.164–0.728); *P *= 0.005] had an independent protective effect on prolonged LOS [OR: 0.307 (95% CI: 0.146–0.648); *P *= 0.002] (Figure 2B). To determine the impact of surgical approach and ERAS protocol combinations on postoperative LOS (Table 4), multivariate analysis was performed to compare the four combinations of ERAS protocol and surgical approach (pre-ERAS + OLR, pre-ERAS + RLR, ERAS + OLR, ERAS + RLR). Compared with the ERAS + RLR strategy, all other surgical approach combinations increased the risk for prolonged LOS.

**Table 4:** Multivariate analysis for prolonged length of stay for patients undergoing liver resection.

	Odds Ratio	95% Confidence Interval	*P*
Pre-ERAS + RLR	4.254	1.340–13.507	**0.014** *
Pre-ERAS + OLR	8.104	2.623–25.031	**0.0001** *
ERAS + OLR	5.195	1.668–16.174	**0.004** *
Age	1.145	1.083–1.210	**0.0001** *
Child-Pugh class	0.166	0.045–0.616	**0.007** *

* Bold values indicate statistically significant *p*-value (*p *< 0.05).

Prolonged length of stay defined as >8 days (median LOS for the pre-ERAS cohort).

### Analysis of the cause of high cost after LR

**Figure 3:**
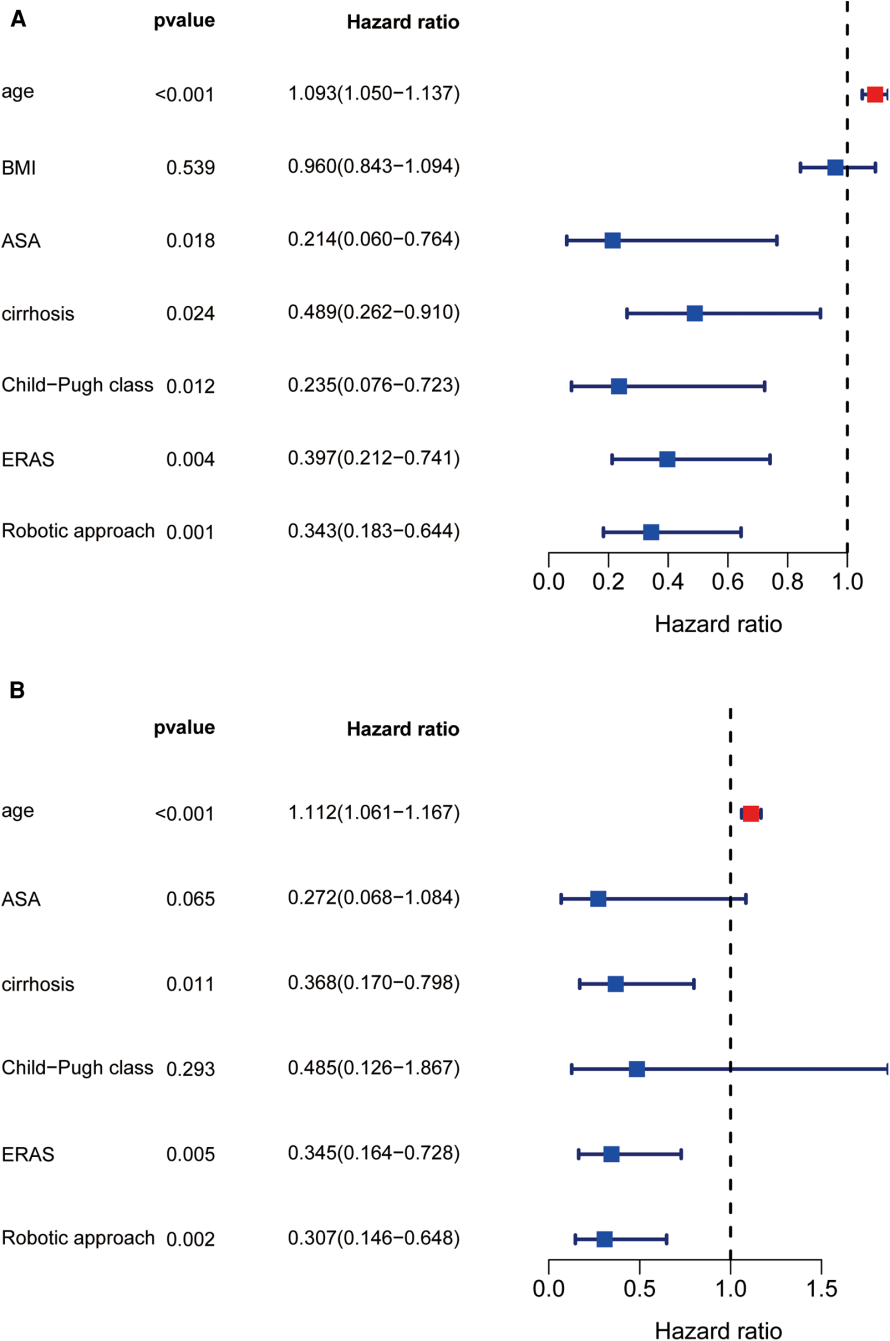
Univariate and multivariate regression analysis about high cost. Forest diagram showed the results of Univariate and multivariate regression analysis (**A,B**).

According to univariate analysis (Figure 3A), factors associated with high total hospitalization costs included increased age, ASA classification, cirrhosis, preoperative Child‒Pugh class B liver function, and non-ERAS protocols. According to multivariate analysis, age [OR: 1.126 (95% CI: 1.071–1.183); *P *< 0.001], ASA classification [OR: 0.282 (95% CI: 0.09–0.885); *P *= 0.03], preoperative cirrhosis [OR: 0.393 (95% CI: 0.187–0.828); *P *= 0.014] and ERAS pathway [OR: 0.47 (95% CI: 0.23–0.961); *P *= 0.038] remained independently associated with a high cost of complicated liver resection (Figure 3B). When comparing the four various combinations of ERAS and operative approach (Table 5), multivariate analysis demonstrated that, compared with ERAS + RLR, the pre-ERAS + RLR cohort was significantly associated with an increased risk for excessive liver resection costs [OR: 8.964 (95% CI: 2.799–28.712); *P *< 0.0001], whereas pre-ERAS + OLR and ERAS + OLR were similar toward increased cost (OR: 1.205 [95% CI: 0.425–3.412]; *P *= 0.726; and OR: 0.785 [95% CI: 0.279–2.205]; *P *= 0.645, respectively).

**Table 5:** Multivariate analysis for high costs for patients undergoing liver resection.

	Odds Ratio	95% Confidence Interval	*P*
Pre-ERAS + RLR	8.964	2.799–28.712	**0.0001** *
Pre-ERAS + OLR	1.205	0.425–3.412	0.726
ERAS + OLR	0.785	0.279–2.205	0.645
Age	1.155	1.091–1.222	**0.0001** *
ASA classification	0.222	0.064–0.776	**0.018** *
Cirrhotic liver	0.437	0.199–0.963	**0.040** *
Child-Pugh class	0.464	0.149–1.439	0.183

Excessive hospital cost defined as >RMB 61,105 (median hospital cost for the pre-ERAS cohort).

* Bold values indicate statistically significant *p*-value (*p *< 0.05).

## Discussion

Liver resection, especially complex hepatectomy, is one of the most difficult abdominal surgeries. Due to the wide length of the excision, complex hepatectomy can result in postoperative liver dysfunction or even failure. Additionally, if the lesion location is special and adjacent to important blood vessels, complex hepatectomy can be associated with difficulties in exposure, which can occur during uncontrolled intraoperative massive bleeding. Circulation and blood supply disorders, such as liver congestion or ischemia, can lead to increased operative difficulty. All of these factors can lead to an increased incidence of postoperative complications, prolonged recovery, LOS, and higher hospitalization costs. As such, faster recovery and postoperative outcome optimization in complex liver resections have received increasing attention.

The application of ERAS protocols in liver surgery as a strategy to fast-track patient recovery has been widely reported. Initial studies showed that the ERAS pathway significantly reduces LOS in patients undergoing hepatectomy without increasing complications (20, 21). Subsequent studies have highlighted the benefits of using ERAS in hepatectomy, with a reduced perioperative stress response, faster recovery of intestinal function, lower pain scores, and shorter LOS (22–25). Summarizing the overall impact of implementing ERAS, a review of more than 1,777 cases of ERAS liver resection and 1,962 cases of traditional care resection revealed that ERAS did not increase mortality or readmission rates but did reduce the risk for prolonged LOS and complications, and significantly reduced hospital costs (26).

The robotic surgical system is ergonomic, with excellent 3D visualizations and wrist instruments. These advantages make liver tumors adjacent to the hilum and major vessels, as well as complex hepatectomy requiring biliary or vascular reconstruction and lymph node dissection, no longer regarded as absolute contraindications.

Several studies have shown that the robotic surgical approach combined with an ERAS pathway further amplifies these benefits. In a prospective cohort study investigating radical prostatectomy, the robotic approach combined with an ERAS pathway reduced the overall cost per patient by 10.8% compared with the control cohort, with faster recovery of urinary incontinence without increasing complications using the robotic approach combined with an ERAS pathway (27). Kowalsky et al*.* demonstrated the additional benefits of the robotic approach combined with the ERAS pathway in a study investigating pancreatic cancer. A comparison of 4 cohorts divided according to the ERAS pathway and surgical approach showed that a combination of ERAS and the robotic approach synergistically decreased LOS and overall cost (8). In two randomized controlled trials of total hysterectomy, postoperative inflammation and tissue damage were lower using the robotic approach with ERAS management than with abdominal hysterectomy, as indicated by high-sensitivity C-reactive protein, white blood cell count, interleukin-6, creatine kinase, and high-mobility group box 1 protein (HMGB1) (28, 29). These results suggest that the ERAS pathway, in combination with the robotic surgical approach, may play a critical role in optimizing perioperative outcomes in complex surgeries.

This analysis demonstrated that implementation of an ERAS pathway can improve the outcomes of open and robotic complex liver resection. Complex liver resection with the ERAS pathway resulted in an earlier return of bowel function, less postoperative pain, shorter LOS, and lower costs. The robotic approach was associated with lower intraoperative blood loss and pain scores, and logistic regression analysis demonstrated that it was protective against prolonged LOS. The robotic approach was, however, associated with an increase in hospital costs, although the use of an ERAS pathway decreased the risk for excessive costs. When compared with the combined strategy of RLR and ERAS, all other combinations resulted in prolonged LOS. More importantly, this combination reduced the incidence of major postoperative complications, suggesting that ERAS and the robotic approach may synergistically optimize the outcomes of complex liver resection.

This study examined the outcomes of complex liver resection-specific ERAS pathways in OLR and RLR. To ensure an intent-to-treat analysis and minimize selection bias, a total of 171 consecutive complex liver resections performed before and after ERAS were included. The size of this cohort enabled a comparison of 4 different ERAS combinations and surgical approaches. In this retrospective study, preoperative clinical data and pathological features were uniformly matched for all 4 combinations. Patients who underwent OLR and RLR benefited from ERAS, and LOS and postoperative hospital costs were reduced in both groups. Similar to the results of laparoscopic hepatectomy, the benefits of the ERAS pathway were most significant in the RLR group. The RLR approach was responsible for increased operative-day costs, which were associated with the increased hospitalization cost per RLR patient compared to OLR. More specifically, the combination of pre-ERAS + RLR had the highest hospitalization costs. These results demonstrated that RLR combined with ERAS could play a synergistic role in reducing increased costs after RLR.

ERAS pathways generally contain elements geared toward limiting the postoperative stress response and accelerating the recovery of physiological function (30). The ERAS protocol described in this report contained many of these components. Preoperative carbohydrate load was associated with a reduced incidence and severity of postoperative nausea/vomiting (31), decreased perioperative insulin resistance, and decreased production of physiological stress markers (32). Similarly, patient-controlled analgesia pumps and subcutaneous injections of bupivacaine have been used to treat postoperative pain due to reduced stress responses (33). Goal-directed restrictive intravenous fluid shifts were used to maintain euvolemia, cardiac output, and delivery of oxygen and nutrients to the tissues, which are important for preserving cellular function, particularly when there is tissue injury and need for repair (34). Urethral catheters and abdominal drainage tubes were withdrawn early to reduce postoperative complications (35). Patient education and engagement are very important. Collaborative discussion regarding ERAS protocol components and desired goals can help improve ERAS compliance and relieve preoperative anxiety. A prospective cohort study involving 436 patients undergoing liver resection reported that higher compliance with ERAS protocols was associated with a lower incidence of major postoperative complications and a shorter postoperative LOS (36). Incorporating these guidelines and evidence-based care components into our ERAS protocols expedited patient recovery and cost savings.

## Conclusion

The results of this study suggest that ERAS implementation improved the outcome of complex hepatectomy. ERAS reduced postoperative LOS and was associated with cost savings in patients undergoing RLR. The robotic approach combined with ERAS minimized LOS and costs compared with other strategies, which may be the optimal combination currently available to control the costs of RLR.

## Data Availability

The raw data supporting the conclusions of this article will be made available by the authors, without undue reservation.
