# Supplementary material for: Robotic approach together with an enhanced recovery programme improve the perioperative outcomes for complex hepatectomy
Source: Front Surg. 2023 Jun 2;10:1135505. doi: 10.3389/fsurg.2023.1135505 (PMC10272522; doi:10.3389/fsurg.2023.1135505)
Supplement: Supplementary file 1 [file Table1.docx]

**Supplemental Table Major components of ERAS liver resection pathway**

|  | **ERAS Pathway** |
| --- | --- |
| Pre-operative management |  |
| Patients Education | -Counseling and introduction to management of ERAS pathway.  -Nutritional status assessment.  -Emphasize functional exercise.  -Prevention of DVT education and risk assessment.  -Smoking cessation. |
| Preparation | -No routine bowel preparation  -Allow free diet, solids oral intake unitil 6 hours before surgery, Clear liquids allowed unitil 2 hours before surgery ( 500ml electrolyte carbohydrate ).  -Avoid oral analgesics and sedatives before surgery. |
| Intra-operative management |  |
| multi-modal anesthesia | -Endotracheal intubation combined with general anesthesia.  -Bupivacaine TAP block under ultrasonographic guidance (bupivacaine 0.25% with max 2.5 mg/kg): 0.25% bupivacaine 1mL/kg or 0.5% bupivacaine 0.5ml/kg.  -Contral central venous pressure during parenchymal transection (CVP < 5 mmHg).  -No routinely nasogastric tube.  -Goal-directed fluid therapy. |
| another | -Routine usage of prophylactic antibiotic.  -Hilar occlusion (<15min).  -Intraoperative prevention of hypothermia (warm blanket, warm saline rinse).  -Minimize the placement of drainage tubes. |
| Postoperative management |  |
|  | -Fluid management: Restricted IV fluids (2000ml), gradually reduce intravenous fluids as the patient eats.  -Pain management: Multimodal analgesia (PCA pump, Subcutaneous bupivacaine /ropivacaine ), POS 1 day removal PCA pump, avoid IV opioids, oral or IV NSAID / APAP .  -Diet management: Drinking 6 hours after surgery, oral liquid or semi-liquid diet POS 1, PONV prevention（Glucocorticoid, metoclopramide), gradually transition to normal diet.  -Functional recovery: Assist flatus and defecation (lactulose, glycerin enema), encourage to mobilization out of bed in the frst 24 hours postoperatively and establish daily activity goals.  -Drainage tube management: POD 1 day removal of Foley catheter, and early removal of abdominal drainage tubes after CT scanning, assess the necessity for invasive intervention which is reduce the readmission rate after discharge.  -In addition to conventional nursing and symptomatic treatment, focus on the treatment of complications ( Postoperative bleeding, liver failure, jaundice, biliary leakage, infection, multiple organ dysfunction) and prevention of deep vein thrombosis ( Conventional low molecular weight heparin and intermittent pneumatic compression device, compression stockings ) |
| Discharge criteria | -Bowel function is restored and normal diet or adequate oral energy intake can be achieved (70% preoperatively);  -No pain or can be controlled well by oral pain relievers;  -Mild complications, no serious complications, or serious complications were cure;  -Liver function test was normal or slightly abnormal, and CT showed no obvious or small amount of peritoneal effusion（<30ml）； |
| Abbreviations: APAP, acetaminophen; CVP, central venous pressure; NSAID, non-steroidal antiinflammatory; PCA, patient-controlled analgesia; PONV, post-op nausea/vomiting; POD, postoperative day | |
