# Supplementary material for: Robotic approach together with an enhanced recovery programme improve the perioperative outcomes for complex hepatectomy
Source: Front Surg. 2023 Jun 2;10:1135505. doi: 10.3389/fsurg.2023.1135505 (PMC10272522; doi:10.3389/fsurg.2023.1135505)
Supplement: Supplementary file 2 [file Table2.docx]

Supplementary Table 2. Detailed costs of liver resection patients.

|  | Total  (n=171) | Pre-ERAS+OLR  (n=42) | Pre-ERAS+RLR  (n=40) | ERAS+OLR  (n=41) | ERAS+RLR  (n=48) | *P* |
| --- | --- | --- | --- | --- | --- | --- |
| Hospital cost (CNY) | 59609(52330-65530) | 58787(49856-63235) | 64334(57968-72458) | 54892(45510-63914) | 58772(52516-63241) | 0.0064* |
| Total intraoperative | 32273(28792-35971) | 28416(24144-31017) | 36296(34279-38994) | 25921(21607-30138) | 33641(31706-36439) | 0.0001* |
| Disposable intraoperative materials | 15998(20463-25438) | 16854(14858-20221) | 26635(20889-28087) | 14928(13246-20362) | 24148(20414-25674) | 0.0001* |
| Nursing care | 2380(1680-3587) | 2650(1880- 3918) | 2384(1588- 3632) | 2436(1545-3689) | 2268(1536-3508) | 0.0006* |
| Other | 26632(22614-30652) | 29615(25691-30666) | 26601(24167-27652) | 25621(22596-30605) | 23571(21568-26158) | 0.0291* |
| * and bold values indicate statistically significant p-value (p < 0.05); Abbreviations: LOS, length of hospital stay; POD, postoperative day; Others cost: including laboratory, radiology, inspection, pathology, housing and and so on. | | | | | | |
